# A prognostic classification system for uveal melanoma based on a combination of patient age and sex, the American Joint Committee on Cancer and the Cancer Genome Atlas models

**DOI:** 10.1111/aos.15210

**Published:** 2022-07-08

**Authors:** Viktor T. Gill, Shiva Sabazade, Christina Herrspiegel, Kathryn G. Ewens, Adrianna Opalko, Nicole Dan, Tinna Christersdottir, Alexander Berg Rendahl, Carol L. Shields, Stefan Seregard, Arupa Ganguly, Gustav Stålhammar

**Affiliations:** ^1^ Department of Pathology Västmanland Hospital Västerås Västerås Sweden; ^2^ Department of Clinical Neuroscience, Division of Eye and Vision Karolinska Institutet Stockholm Sweden; ^3^ St. Erik Eye Hospital Stockholm Sweden; ^4^ Department of Genetics, Perelman School of Medicine University of Pennsylvania Philadelphia Pennsylvania USA; ^5^ Ocular Oncology Service, Wills Eye Hospital Thomas Jefferson University Philadelphia Pennsylvania USA

**Keywords:** ciliary body melanoma, histology, monosomy 3, oncology, pathology, prognosis, symptoms, uveal melanoma, vasculogenic mimicry

## Abstract

**Purpose:**

To revisit the independent importance of ciliary body involvement (CBI), monosomy 3 (M3), tumour size, histological and clinical factors in uveal melanoma (UM) and to devise a new prognostic classification based on a combination of the American Joint Committee on Cancer (AJCC) and the Cancer Genome Atlas (TCGA) models.

**Methods:**

Two cohorts with a total of 1796 patients were included. Clinicopathological factors were compared between patients with and without CBI and M3. Development of the prognostic classification was performed in a training cohort and was then tested in two independent validation cohorts.

**Results:**

Tumours with CBI were more common in women, had greater apical thickness, greater basal tumour diameter, greater rates of vasculogenic mimicry and greater rates of M3, were more often asymptomatic at diagnosis and had poorer 5‐ and 10‐year globe conservation rates (*p* < 0.023). In multivariate logistic regression, patient age at diagnosis, tumour diameter and CBI were independent predictors of M3 (*p* < 0.001). In multivariate Cox regression, male sex, age at diagnosis, tumour diameter, M3 and CBI were independent predictors of metastasis. The proposed prognostic classification combined patient age, sex, CBI, extraocular extension, M3, 8q (optional) and tumour size, and demonstrated greater prognostic acumen than both AJCC 4 T categories and TCGA groups A to D in validation cohorts.

**Conclusions:**

Tumour size does not confound the prognostic implication of CBI, M3, male sex and age at diagnosis in UM. These factors were included in a new prognostic classification that outperforms AJCC T category and TCGA groups.

## 1 INTRODUCTION

Uveal melanomas (UMs) most commonly arise in the choroid (90% of cases), followed by the ciliary body (6%) and iris (4%) (Shields et al., 2009). Whereas iris melanomas are distinguished by their relatively favourable prognosis and genomic features associated with ultraviolet radiation damage, choroidal and ciliary body melanomas have a higher propensity for metastatic spread and are likely initiated by punctuated evolution oncogenic events that are not strongly associated with sunlight exposure (Jager et al., 2020; Johansson et al., 2020).

Ciliary body involvement (CBI) is associated with an increased risk for metastasis, with about one‐third of patients developing the metastatic disease within 10 years of diagnosis (Shields et al., 2009). The specific reason for the worse prognosis remains unclear but has previously been attributed to an intrinsic tumour “aggressiveness” (Berus et al., 2017; Schmitzer et al., 2016; Torossian et al., 2015) or “malignant” behaviour (McLean et al., 1982), or by a tendency for accumulation of risk factors (Kaliki & Shields, 2017; McLean et al., 1982; Rummelt et al., 1995). Other explanations have also pointed to the continuous contractions of the ciliary muscle as a causal factor in tumour spread or to the exceptionally rich vascularization of this region (Costache et al., 2013; Kaliki & Shields, 2017). The unfavourable prognostic association has also been related to the predilection for monosomy 3 (M3) and gain of 8q, as well as tumour microvascular patterns (Damato & Coupland, 2009; McLean et al., 1982; Prescher et al., 1996; Rummelt et al., 1995).

For more than 25 years, M3 has been known as one of the strongest prognostic factors in UM (Prescher et al., 1990; Sisley et al., 1990; Horsman & White, 1993; Prescher et al., 1996; Shields et al., 2017a, 2017b). Further, partial loss of chromosome 3 and mosaicism have more recently been associated with shorter metastasis‐free survival (Rodrigues et al., 2020; Vaquero‐Garcia et al., 2017). The latter refers to a pattern of mixed chromosome 3 disomy and monosomy, likely as a result of tumour heterogeneity. In turn, M3 is associated with virtually all other strong predictors: larger tumour size (Shields et al., 2007), *BAP1* mutation (Harbour et al., 2010), gene expression class 2 (Onken et al., 2004; Worley et al., 2007), vasculogenic mimicry patterns (Meir et al., 2007), extraocular extension (van Beek et al., 2014) and tumour cell morphology (Scholes et al., 2003; Sisley et al., 1997). A current understanding of the sequence of molecular events in a growing, metastasizing UM is that mutations in the G_q/11_ pathway are necessary for clonal expansion of tumour cells, whereas near‐mutually exclusive mutations in *BAP1*, *SF3B1* or *EIF1AX* confer malignant and metastatic potential (Harbour et al., 2010, 2013; Martin et al., 2013; Onken et al., 2008; Van Raamsdonk et al., 2009, 2010). M3 may represent the second hit in bi‐allelic loss of *BAP1*, but occasionally, *BAP1* mutations have also been observed without loss of heterozygosity (Field et al., 2018). Although the correlation between M3 and the other risk factors is not without exceptions, it is a useful marker for aggressive disease. In a more recent model based on results from the Cancer Genome Atlas (TCGA) project, chromosome 3 status is combined with the gain of one or multiple copies of 8q to classify patients into four categories (groups A to D) with increasing metastatic risk (Jager et al., 2018; Mazloumi et al., 2020; Robertson et al., 2017).

In several multivariate models that have included basal area or tumour diameter as covariates, ciliary body origin or involvement has been another independent predictor of poor survival. However, these models have often not included tumour thickness, which is an important prognostic indicator in UM (Augsburger & Gamel, 1990; Li et al., 2000; Rummelt et al., 1995; Seddon et al., 1983; Shields et al., 2009). One exception is a large study by Kujala et al. (2013), who found that tumour diameter, tumour thickness, CBI and extraocular extension were all independent predictors of melanoma‐related mortality in multivariate Cox regression (Kujala et al., 2013). In contrast to iris melanomas who have no better prognosis than choroidal melanomas when adjusting for tumour diameter and thickness, this should make it unlikely that increased tumour size is the real reason for the prognostic implication of CBI (Johansson et al., 2020; Sabazade et al., 2021a, 2021b). The American Joint Committee on Cancer (AJCC) classification of UM consequently assigns tumours with CBI a subcategory with shorter metastasis‐free survival (Kujala et al., 2013; Kujala & Kivela, 2005; Simpson et al., 2015). Other prognostic factors include patient sex and presenting symptoms (Fili et al., 2020; Park et al., 2017; Zloto et al., 2013). In a recent publication, it was found that AJCC stage II and III modifies the risk of metastasis in TCGA groups C and D, but patient sex and age were not included in the analysis (Gelmi et al., 2022).

In this study, we propose a new prognostic point system that integrates the AJCC classification, the TCGA model as well as additional clinical factors. To clarify the independent prognostic importance of CBI, tumour size, patient age, sex and M3, we first examine the relationship between these factors, as well as the symptoms and histological characteristics of patients and tumours with and without CBI. For example, if the prognostic implication of CBI was not independent of M3, both were not to be included in the prognostic system.

## 2 METHOD

### 2.1 Patient selection

This study was approved by the Swedish Ethical Review Authority (reference 2020‐02835) and adhered to the tenets of the Declaration of Helsinki.

Firstly, all patients who had been treated for choroidal or ciliary body melanoma at St. Erik Eye Hospital, Stockholm, Sweden after 1 January 1995, were considered for the study (cohort 1). Retrospective clinicopathological data including presenting symptoms, tumour size, treatment modality, treatment failure and cause of death was retrieved from a treatment registry. Out of 1987 patients, 1295 were excluded as they did not have detailed information about ciliary body status available (location of tumour origin, the presence or absence of CBI, AJCC T category). These 1295 excluded patients had similar metastasis‐free survival as the included patients, as reported in the descriptive statistics section below. A further 20 patients were excluded because they had tumours that originated in the iris. Six hundred and seventy‐two patients remained for analysis. Informed consent had been obtained from all patients before inclusion in the treatment registry. According to the approved ethics application, the requirement for written informed consent for this particular study was however waived because this was a retrospective study that does not affect the treatment or follow‐up of the patients. Further, all patient data had been previously collected and no new clinical data collection was performed, and no new tissues were sectioned, stained or otherwise processed. As no data on chromosome 3 status was available for the Swedish patients, we also included a second cohort of 1227 UMs previously published by Vaquero‐Garcia et al. (cohort 2) (Vaquero‐Garcia et al., 2017). Thirty‐five patients were excluded because they had tumours that originated in the iris. Sixty‐eight patients were excluded because their chromosome 3 status was not available. Eleven hundred and twenty‐four patients remained for analysis. Informed consent for the use of excess tissue and relevant information for research purposes had been obtained from all individuals who submitted samples for chromosomal testing.

For all patients, tumour dimensions, location and CBI (yes or no) had been determined by examination with A‐ and B‐scan ultrasonography, ultrasound biomicroscopy and/or slit‐lamp biomicroscopy at the time of initial diagnosis. The *extent* of CBI was not available for any included patient, that is, how much of the ciliary body was infiltrated by tumour. The STROBE cross‐sectional reporting guidelines were used (von Elm et al., 2007). A summary of what data was included from which cohort is provided in given in Table S1.

#### 2.1.1 Vasculogenic mimicry

In 116 enucleated eyes from cohort 1, patterns of microvascular loops and networks were assessed in 4 μm tissue sections stained with Periodic‐acid Schiff (PAS) without haematoxylin counterstain. These sections had been used for diagnostic purposes and were available in the archive of the St. Erik Ophthalmic Pathology Laboratory. To fulfil regulatory requirements, no formalin‐fixed paraffin‐embedded or fresh biobank tissue from living patients was collected, sectioned, stained or otherwise processed. A light microscope with a green narrow band pass filter was used, according to the method described by Folberg et al. (1992). Nine patterns were evaluated: normal, silent, straight, parallel, parallel with crosslinks, arcs, arcs with branches, closed loops and networks. The presence of one or several of the patterns within any given tumour was recorded. Since the association of some of these patterns has been described as having a particularly strong correlation with the development of metastases in UM, tumours with and without CBI were compared for the presence of networks, closed loops or arcs with branching, or any combination thereof using a previously published method (McLean et al., 1997; Seregard et al., 1998; Makitie et al., 1999; Stalhammar et al., 2019a, 2019b).

### 2.2 Chromosomal analysis

Archived and fresh tumour samples collected following enucleation or fine‐needle aspiration biopsy of tumours were submitted to the Genetic Diagnostic Laboratory, University of Pennsylvania, Philadelphia, PA, USA. Chromosomal copy number analysis using whole‐genome single‐nucleotide polymorphism arrays was performed on Cohort 2 samples as previously described (Ewens et al., 2013; Vaquero‐Garcia et al., 2017).

### 2.3 Statistical analysis

*p*‐Values below 0.05 were considered statistically significant, all *p*‐values being two‐sided. For tests of continuous variables that did not deviate significantly from normal distribution (Shapiro–Wilk test *p* > 0.05) Student's *t*‐tests were used. For non‐parametrical data, Mann–Whitney *U* tests were used. In comparisons of categorical variables, we used two‐by‐two contingency tables and Pearson chi‐square (χ^2^) tests (if all fields had a sample of >5) or Fisher's exact tests (if any field had a sample of <5). Linear trends for the proportion of tumours with M3 with increasing tumour diameter, thickness and volumes were calculated, and differences in the linear trend between tumours with and without CBI were compared through analysis of covariance (ancova). The volume of tumours was estimated assuming a semi‐ellipsoid shape (Uner et al., 2021):

$$\text{Volume of tumour}=\frac{\pi }{6}\times t\times {\mathrm{lbd}}^{2}$$

Uni‐ and multivariate binary logistic regressions were used to examine the relationship between patient sex, age, tumour size, CBI and M3. For comparisons of association with metastasis, multivariate Cox regression hazard ratios (HR) were calculated. To test whether our data met the proportional hazard assumption, we built a Cox regression model to calculate the HR for UM‐related mortality with a time‐dependent versus a time‐independent variable (AJCC T category) as covariates. As the exact date of metastasis could only be approximated within a few weeks for some patients, survival curves were generated by the actuarial life table method in 6‐month intervals and the Wilcoxon (Gehan) test was applied. In the development of a prognostic point system, patients were randomly assigned to either a training or a validation cohort, in a 1:1 ratio. The final system designated each patient into one of four prognostic groups, based on the relative weight of prognostic factors in the training cohort. If no established cut‐offs were available for continuous variables, receiver operating characteristics (ROC) were analysed with emphasis on specificity for metastasis. The final prognostic point system was then tested on the validation cohort. The relative prognostic acumen of this system to AJCC T categories 1–4 was evaluated with uni‐ and multivariate Cox regression HRs and the Wald statistic, as well as with ROC. We also compared the prognostic point system to the four groups in the TCGA system (group A: disomy 3 and normal 8q; group B disomy 3 and any 8q gain; group C M3 and three copies of 8q; group: D M3 with >3 copies of 8q) in the TCGA cohort of 80 tumours (Robertson et al., 2017). All statistical analyses were performed using IBM SPSS statistics version 27 (Armonk, NY, USA) and GraphPad Prism version 9.3.0 (San Diego, CA, USA).

## 3 RESULTS

### 3.1 Descriptive statistics

Of the 1796 included patients, 882 (49%) were female and 914 (51%) were male. Their mean age at diagnosis was 60 years (SD 15). The mean tumour largest basal diameter was 11.6 mm (SD 4.3) and thickness 5.5 mm (SD 3.2). Two hundred and ninety‐nine tumours (17%) involved the ciliary body and 1497 (83%) did not. Three hundred and fifty‐three patients developed metastases before the end of follow‐up. The median follow‐up for the 1443 patients that did not develop metastases was 3.8 years (IQR 3.5, Table 1). Five hundred and ninety‐six of 1124 tumours (53%) with chromosome 3 status available had loss of heterozygosity. The data met the proportional hazards assumption (*p* = 0.55). There was no significant difference in metastasis‐free survival between the 672 included and 1295 excluded patients from cohort 1 (Wilcoxon (Gehan) *p* = 0.12, Figure S1).

**TABLE 1:** Demographics and clinical features of study patients and tumours

	Both cohorts	Cohort 1	Cohort 2	*p*
*n* =	1796	672	1124	
CBI, *n* (%)				<0.001
No	1497 (83)	593 (88)	904 (80)	
Yes	299 (17)	79 (12)	220 (20)	
Mean age at diagnosis, years (SD)	60 (15)	64 (14)	57 (14)	<0.001
Sex, *n* (%)				0.04
Female	882 (49)	309 (46)	573 (51)	
Male	914 (51)	363 (54)	551 (49)	
Mean tumour thickness, mm (SD)	5.5 (3.2)	5.4 (2.7)	5.6 (3.4)	0.92
Mean tumour diameter, mm (SD)	11.6 (4.3)	11.3 (4.2)	11.8 (4.4)	0.027
AJCC T category, *n* (%)				<0.001
1a	560 (31)	383 (33)	177 (29)	
1b	48 (3)	31 (3)	17 (3)	
1c	11 (<1)	0 (0)	11 (2)	
2a	440 (25)	289 (25)	151 (24)	
2b	60 (3)	48 (4)	12 (2)	
2c	19 (1)	0 (0)	19 (3)	
3a	275 (15)	200 (17)	75 (12)	
3b	125 (7)	99 (8)	26 (4)	
3c	8 (<1)	0 (0)	8 (1)	
4a	105 (6)	80 (7)	25 (4)	
4b	56 (3)	44 (4)	12 (2)	
N/a	89 (5)	3 (<1)	86 (14)	
Primary treatment modality				
Plaque brachytherapy		500 (74)		
Enucleation		172 (26)		
Chromosome 3 status, *n* (%)				
Disomy			528 (47)	
Monosomy 3, *n* (%)			386 (34)	
Partial monosomy 3, *n* (%)			106 (9)	
Mosaicism, *n* (%)			104 (9)	
Follow‐up years, median^a^ (IQR)	3.8 (3.5)	4.0 (3.8)	3.7 (3.4)	

Abbreviations: CBI, Ciliary body involvement; IQR, Interquartile range; SD, Standard deviation.

^a^ For metastasis‐free patients.

### 3.2 Patient and tumour characteristics

As seen in Table 2, tumours with CBI were more common in women (χ^2^ *p* < 0.002), had greater apical thickness (7.71 vs. 5.04 mm, Student's *t*‐test *p* < 0.001), greater basal tumour diameter (13.66 vs. 11.23 mm, *p* < 0.001), greater rates of vasculogenic mimicry (35 vs. 13%, χ^2^ *p* = 0.023) and greater rates of M3 (55 vs. 29%, χ^2^ *p* < 0.001). Conversely, tumours with CBI had significantly lower rates of retinal detachment (39 vs. 53%, χ^2^ *p* < 0.001).

**TABLE 2:** Characteristics of tumours with and without CBI

In both cohorts (*n* = 1796)	No CBI (*n* = 1497)	CBI (*n* = 299)	*p*
Sex, *n* (%)			
Female	711 (47)	171 (57)	0.002
Male	786 (53)	128 (43)	
Age at diagnosis, mean years (SD)	59.3 (14.4)	60.6 (15.1)	0.18
Tumour size, mean (SD)			
Thickness, mm	5.04 (3.02)	7.71 (3.08)	<0.001
Diameter, mm	11.23 (4.13)	13.66 (4.63)	<0.001

In cohort 1 (*n* = 672)	No CBI (*n* = 593)	CBI (*n* = 79)	
Retinal detachment^†^, *n* (%)	313 (53)	31 (39)	0.024
Treatment modality, *n* (%)			
Plaque brachytherapy	442 (75)	58 (73)	0.83
Enucleation	151 (26)	21 (27)	

In the 116 enucleated tumours	No CBI (*n* = 99)	CBI (*n* = 17)	
Vasculogenic mimicry*, *n* (%)	13 (13)	6 (35)	0.023
Tumour cell type, *n* (%)			
Epithelioid	26 (26)	3 (18)	0.057
Mixed	54 (55)	14 (82)	
Spindle	19 (19)	0 (0)	

In cohort 2 (*n* = 1124)	No CBI (*n* = 904)	CBI (*n* = 220)	
Chromosome 3 status, *n* (%)			
Disomy	469 (52)	59 (27)	
Monosomy 3, *n* (%)	266 (29)	120 (55)	<0.001
Partial monosomy 3, *n* (%)	86 (10)	20 (9)	
Mosaicism, *n* (%)	83 (9)	21 (10)	

Abbreviations: CBI, Ciliary body involvement; SD, Standard deviation.

^**†**^ Clearly visible upon slit‐lamp examination, involving a significant proportion of the retina beyond the tumour base.

* Presence of vasculogenic mimicry defined as networks, closed loops or arcs with branching, or any combination thereof as these patterns have been shown to be the most prognostically relevant.

There were however no significant differences in patients' age at diagnosis (based on *n* = 1796), treatment modality (*n* = 672) or tumour cell types (*n* = 116) between tumours with and without CBI (*p* ≥ 0.057, Table 2).

**FIGURE 1:**
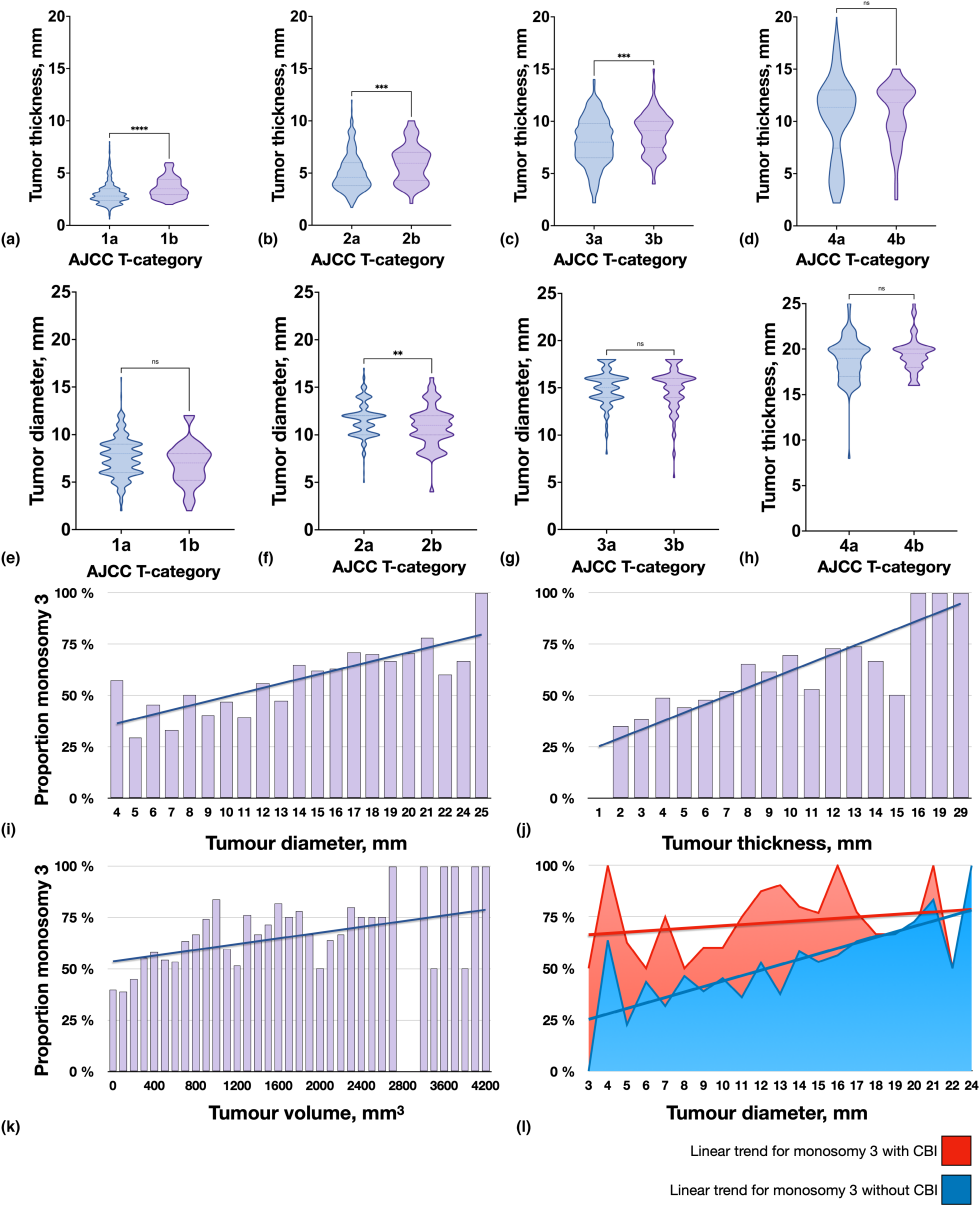
Tumour size across American Joint Committee on Cancer (AJCC) T subcategories a and b, and proportion of M3 tumours with increasing tumour size. (a) Tumour thickness within T category 1a and 1b, (b) 2a and 2b, (c) 3a and 3b and (d) 4a and 4b. (e) Tumour diameter within T category 1a and 1b, (f) 2a and 2b, (g) 3a and 3b, and (h) 4a and 4b. (i) Proportion of tumours with M3 across tumour diameters. (j) Proportion of tumours with M3 across tumour thicknesses. (k) Proportion of tumours with M3 across tumour volumes. The blue lines indicate linear trends. (l) Tumours with CBI (yellow field) had a larger proportion of M3 at small tumour diameters, but the linear trend for the proportion of tumours with M3 as a function of tumour diameter had a steeper slope for tumours without CBI (2.2 vs. 0.6 percentage points per increasing mm, ancova *p* = 0.003). *p < 0.05; **p < 0.01; ***p < 0.001; ****p < 0.0001. Ns, non‐significant.

When only comparing tumours from the same American Joint Committee on Cancer (AJCC) T category, T1b, T2b and T3b tumours had greater apical thickness than T1a, T2a and T3a tumours, respectively (Mann–Whitney *U p* < 0.001, Figure 1a–c). T4b and T4a tumours had similar thicknesses (*p* = 0.86, Figure 1d). There were no significant differences in tumour diameter between T1a, T3a or T4a and T1b, T3b or T4b tumours, respectively, but T2b tumours had a significantly smaller diameter than T2a tumours (*p* = 0.006, Figure 1e–h).

When also including Tc and Td categories, the distributions of tumour diameters were dissimilar in the T1, T2 and T4 categories and the distributions of tumour apical thickness dissimilar in the T1, T2 and T3 categories. In all available comparisons, tumours with extraocular extension ≤5 mm (Tc tumours) had greater diameter and smaller thickness than tumours with CBI (Tb tumours, Table 3).

**TABLE 3:** Distribution of tumour sizes over AJCC T‐categories

	Mean diameter, mm (SD)	*p* *	Mean thickness, mm (SD)	*p* *
T‐category, (*n*)				
1a, (560)	7.53 (2.00)	0.036	3.02 (1.02)	<0.001
1b, (51)	6.91 (2.23)		3.65 (1.02)	
1c, (11)	8.82 (2.44)		3.41 (1.01)	
1d, (0)				
2a, (440)	11.61 (1.77)	0.027	5.00 (1.73)	0.001
2b, (63)	10.87 (2.18)		5.94 (1.85)	
2c, (19)	11.37 (2.45)		5.33 (2.19)	
2d, (0)				
3a, (275)	15.11 (1.84)	0.69	7.95 (2.27)	<0.001
3b, (127)	14.90 (2.25)		8.86 (1.93)	
3c, (8)	15.71 (1.25)		6.95 (2.58)	
3d,				
4a, (105)	19.25 (3.84)	<0.001	11.22 (6.04)	0.95
4b, (58)	19.50 (2.22)		10.79 (2.92)	
4c, (0)				
4d, (0)				

AJCC, American Joint Committee on Cancer; SD, Standard deviation; N/a, not available.

* Mann–Whitney *U* test if 2 groups, Kruskal–Wallis test if >2 groups.

With increasing tumour diameter, thickness, or volume, a gradually larger proportion of tumours had M3 (Figure 1i–k). A larger proportion of tumours with CBI had M3 at small diameters. Among tumours with a diameter of <10 mm, 26 of 41 tumours (63%) with CBI and 136 of 344 tumours (40%) without CBI had M3. Among tumours with a diameter of ≥18 mm, 38 of 54 tumours (70%) with CBI and 62 of 91 tumours (68%) without CBI had M3. The linear trend for the proportion of tumours with M3 as a function of tumour diameter had a steeper slope for tumours without CBI (2.2 vs. 0.6 percentage points per increasing mm, ancova *p* = 0.003, Figure 1l).

### 3.3 Symptoms, treatment modality and risk for treatment failure

Among the 672 patients in cohort 1, patients that had tumours with CBI were more often asymptomatic at diagnosis (χ^2^ *p* = 0.02), but there were no differences in the proportion of patients that presented with blurry vision (*p* = 0.37), a shadow in the visual filed (*p* = 0.11), a painful eye (*p* = 0.59), metamorphopsia (*p* = 0.78) or flashes and/or floaters (*p* = 0.70). The mean symptom duration before diagnosis for patients with tumours with and without CBI was 3.3 and 4.0 months, respectively (Mann–Whitney *U p* = 0.18).

**FIGURE 2:**
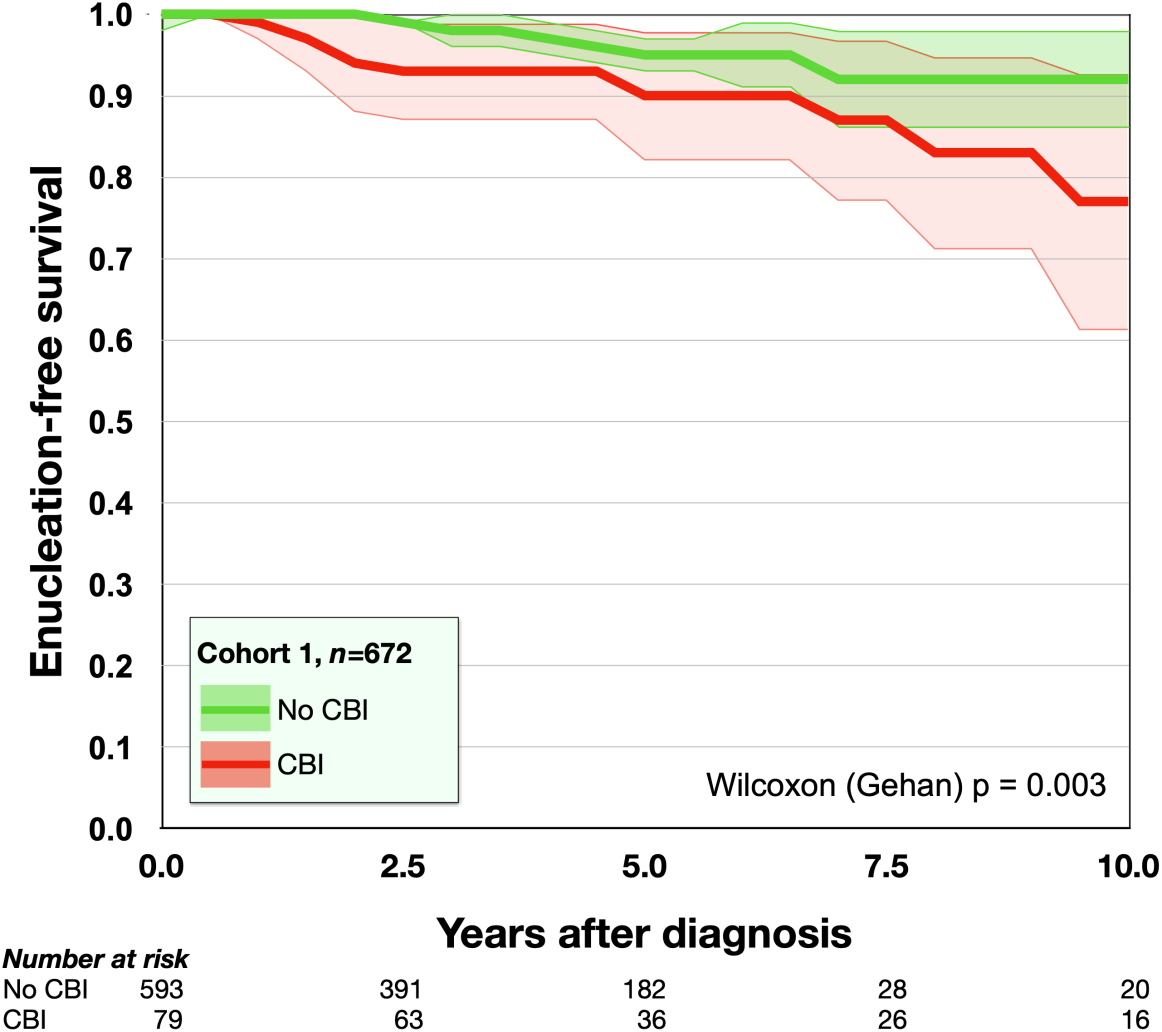
Cumulative enucleation‐free survival after plaque brachytherapy of uveal melanomas with (red) and without (green) ciliary body involvement. CBI, ciliary body involvement.

Patients with and without CBI were treated similarly, with 21 of 79 (27%) and 151 of 593 (25%) being primary enucleated and the rest receiving plaque brachytherapy, respectively (χ^2^ *p* = 0.83). Out of 58 patients with CBI treated with plaque brachytherapy, 11 (19%) had to undergo secondary enucleation, which was a significantly greater proportion than the 15 of 442 patients without CBI (3%, *p* < 0.001). The 5‐ and 10‐year cumulative globe conservation rate was 95% and 92% for patients without CBI, versus 90% and 77% for patients with CBI (Wilcoxon (Gehan) *p* = 0.003, Figure 2). In multivariate Cox regression with tumour thickness and diameter entered as covariates, CBI was not an independent predictor of secondary enucleation (HR 2.0, 95% CI 0.7–5.6, *p* = 0.20).

### 3.4 Regression analyses

In univariate binary logistic regression, patient age at diagnosis (OR 1.18 per increasing decade, 95% CI 1.09–1.28, *p* < 0.001), tumour diameter (OR 1.09 per increasing mm, 95% CI 1.06–1.12, *p* < 0.001), tumour thickness (OR 1.13 per increasing mm, 95% CI 1.09–1.17, *p* < 0.001) and CBI (OR 3.01, 95% CI 2.18–4.16, *p* < 0.001) were associated with M3.

In multivariate binary logistic regression, patient age at diagnosis (OR 1.18 per increasing decade, 95% CI 1.09–1.29, *p* < 0.001), tumour diameter (OR 1.05 per increasing mm, 95% CI 1.01–1.10, *p* < 0.001) and CBI (OR 2.21, 95% CI 1.56–3.13, *p* < 0.001) retained their significance (Table 4).

**TABLE 4:** Binary logistic regression

	B	S.E.	Wald	*p*	Exp(B)	95% CI lower	95% CI upper
Univariate							
Male sex	−0.04	0.12	0.09	0.76	0.97	0.77	1.21
Age at diagnosis^a^	0.17	0.04	17.42	<0.001	1.19	1.10	1.29
Tumour diameter, mm^b^	0.09	0.01	37.82	<0.001	1.09	1.06	1.12
Tumour thickness, mm^b^	0.12	0.02	35.92	<0.001	1.13	1.09	1.17
CBI	1.10	0.17	44.62	<0.001	3.01	2.18	4.16
Multivariate							
Age at diagnosis^a^	0.17	0.04	15.23	<0.001	1.18	1.09	1.29
Tumour diameter, mm^b^	0.05	0.02	7.05	0.008	1.05	1.01	1.10
Tumour thickness, mm^b^	0.05	0.03	2.92	0.087	1.05	0.99	1.10
CBI	−1.94	0.33	34.45	<0.001	2.21	1.56	3.13

*Note*: Dependent variable: Chromosome 3 monosomy.

^a^ Per increased decade.

^b^ Per increasing mm. Loss of chromosome 3 heterozygosity is defined as monosomy, partial monosomy or mosaicism. CBI, Ciliary body involvement. Based on 1124 cases from cohort 2.

In univariate Cox regression, male sex (HR 1.58, 95% CI 1.27–1.95, *p* < 0.001), patient age at diagnosis (HR 1.18 per increased decade, 95% CI 1.09–1.28, *p* < 0.001), tumour diameter (HR 1.12 per increased millimetre, 95% CI 1.19–1.24, *p* < 0.001), tumour thickness (HR 1.18 per increased millimetre, 95% CI 1.16–1.20, *p* < 0.001), M3 (HR 4.18, 95% CI 3.04–5.74, *p* < 0.001) and CBI (HR 2.31, 95% CI 1.84–2.90, *p* < 0.001) were all significant predictors of metastasis.

In multivariate Cox regression, male sex (HR 1.70, 95% CI 1.27–2.28, *p* < 0.001), patient age at diagnosis (HR 1.19, 95% CI 1.07–1.32, *p* = 0.001), tumour diameter (HR 1.22. 95% CI 1.17–1.28, *p* < 0.001), M3 (HR 4.31, 95% CI 2.89–6.41, *p* < 0.001) and CBI (HR 1.62, 95% CI 1.19–2.20, *p* = 0.002) retained their significance (Table 5).

**TABLE 5:** Cox regression, hazard for metastasis

	B	S.E.	Wald	*p*	Exp(B)	95% CI lower	95% CI upper
Univariate							
Male sex	0.46	0.11	17.34	<0.001	1.58	1.27	1.95
Age at diagnosis^a^	0.16	0.04	15.65	<0.001	1.18	1.09	1.28
Tumour diameter, mm^b^	0.19	0.01	404.24	<0.001	1.12	1.19	1.24
Tumour thickness, mm^b^	0.17	0.01	238.54	<0.001	1.18	1.16	1.20
Monosomy 3	1.43	0.16	78.07	<0.001	4.18	3.04	5.74
CBI	0.84	0.12	51.95	<0.001	2.31	1.84	2.90
Multivariate							
Male sex	0.53	0.15	12.81	<0.001	1.70	1.27	2.28
Age at diagnosis^a^	0.18	0.05	11.03	0.001	1.19	1.07	1.32
Tumour diameter, mm^b^	0.20	0.02	78.40	<0.001	1.22	1.17	1.28
Tumour thickness, mm^b^	0.01	0.03	0.01	0.94	1.00	0.95	1.05
Monosomy 3	1.46	0.20	51.86	<0.001	4.31	2.89	6.41
CBI	0.48	0.16	9.59	0.002	1.62	1.19	2.20

^a^ Per increased decade.

^b^ Per increasing mm. CBI, Ciliary body involvement. Based on 1124 cases from cohort 2.

### 3.5 Metastasis‐free survival

**FIGURE 3:**
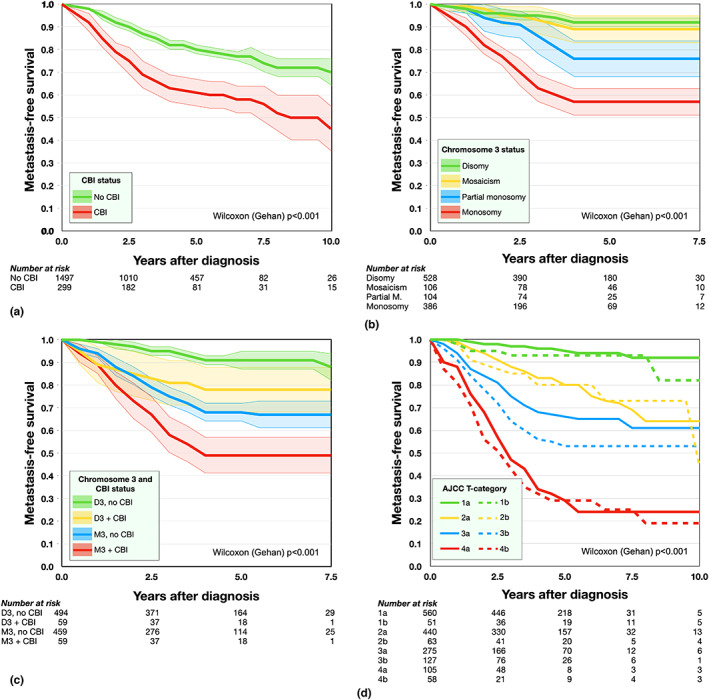
Metastasis‐free survival according to ciliary body involvement (CBI), chromosome 3 status, and AJCC T category. (a) Patients with CBI (red) had significantly shorter metastasis‐free survival. (b) Monosomy 3 (M3, red) entailed a shorter metastasis‐free survival than partial M3 (blue), which in turn entailed a shorter survival than mosaicism (yellow), which in turn entailed a shorter survival than disomy 3 (green). (c) M3 and CBI (red) entailed a shorter metastasis‐free survival than M3 without CBI (blue), which in turn entailed a shorter survival than disomy 3 and CBI (yellow), which in turn entailed a shorter survival than disomy 3 without CBI (green). (d) Tumours with CBI (dashed lines) had slightly shorter metastasis‐free survival within each AJCC T category.

Patients with tumours that involved the ciliary body had significantly shorter metastasis‐free survival (Wilcoxon (Gehan) *p* < 0.001, Figure 3a). M3 entailed a shorter metastasis‐free survival than partial M3, which in turn entailed a shorter survival than mosaicism, which in turn entailed a shorter survival than disomy 3 (*p* < 0.001, Figure 3b). M3 and CBI entailed a shorter metastasis‐free survival than M3 without CBI, which in turn entailed a shorter survival than disomy 3 and CBI, which in turn entailed a shorter survival than disomy 3 without CBI (*p* < 0.001, Figure 3c). As expected, tumours with CBI had slightly shorter metastasis‐free survival within each AJCC T category (*p* < 0.001, Figure 3d).

### 3.6 Prognostic point system

With the aim to establish a four‐tiered classification that improves on current systems and identifies patients with very high, intermediate and very low risk for metastases, we devised a prognostic point system that combines AJCC T categories 1–4, TCGA groups A to D and the additional clinical factors examined herein (patient age and sex).

To obtain relative prognostic point weights for patient sex, patient age at diagnosis, AJCC T category, M3 and CBI, and to avoid the risk of overfitting, our sample was randomized into one training cohort (*n* = 899) and one validation cohort (*n* = 897). Considering that data on M3 was missing from cohort 1, those patients were list‐wise excluded from further analysis. In multivariate Cox regression in the training cohort, male sex, patient age at diagnosis, AJCC T category, M3 and CBI were confirmed to be independent predictors of metastasis (Table S2). One half prognostic point was assigned to each male sex and CBI, one full point was assigned to each increasing AJCC T category (i.e., T category 1: One point; 2: Two points; 3: Three points and 4: Four points) and two points were assigned to M3. An extraocular extension was rare in our cohorts (38 of 1796 patients, 2%), why extraocular extension ≤5 mm was given one and a half point, and >5 mm three points based on previous extensive evidence of the prognostic importance of these factors (Kujala et al., 2013). The Cancer Genome Atlas group C is characterized by M3 and three copies of 8q, and group D by M3 and >three copies of 8q. When this data are available, one prognostic point can therefore be given to the former and two to the latter. To obtain an appropriate cut‐off level for patient age at diagnosis (a continuous variable) we proceeded with ROC of patient age with emphasis on >75% specificity for metastasis in the training cohort. Patient age of 70 years at diagnosis yielded a specificity of 77% and a sensitivity of 35% (Figure S2). One‐‐half prognostic point was therefore given to patients that were over 70 years at diagnosis.

Patients could thereby be given a total of 0–10.5 points (if data on 8q copy number is not available) or 0–12.5 points (if data on 8q copy number is available), which was divided into four prognostic groups (Table 6).

**TABLE 6:** Prognostic group classification [Correction added on 14 July 2022, after first online publication: The subtitles for Table 6 and the last row were included in Table 6B in this current version.]

(A) Chromosome 8q copy number not available		(B) Chromosome 8q copy number available	
Factor	Points	Factor	Points
Male sex	0.5	Male sex	0.5
Age over 70	0.5	Age over 70	0.5
CBI	0.5	CBI	0.5
Extraocular extension ≤5 mm	1.5	Extrascleral growth ≤5 mm	1.5
Extraocular extension >5 mm	3	Extrascleral growth >5 mm	3
Monosomy 3	2	Monosomy 3	2
AJCC T‐category		3 copies of 8q	1
1	1	>3 copies of 8q	2
2	2	AJCC T‐category	
3	3	1	1
4	4	2	2
		3	3
		4	4
Prognostic group 1	≤2 points	Prognostic group 1	≤2 points
Prognostic group 2	2.5–4 points	Prognostic group 2	2.5–4.5 points
Prognostic group 3	4.5–6 points	Prognostic group 3	5–7 points
Prognostic group 4	≥6.5 points	Prognostic group 4	≥7.5 points

CBI, Ciliary body involvement. M3, Monosomy 3.

**FIGURE 4:**
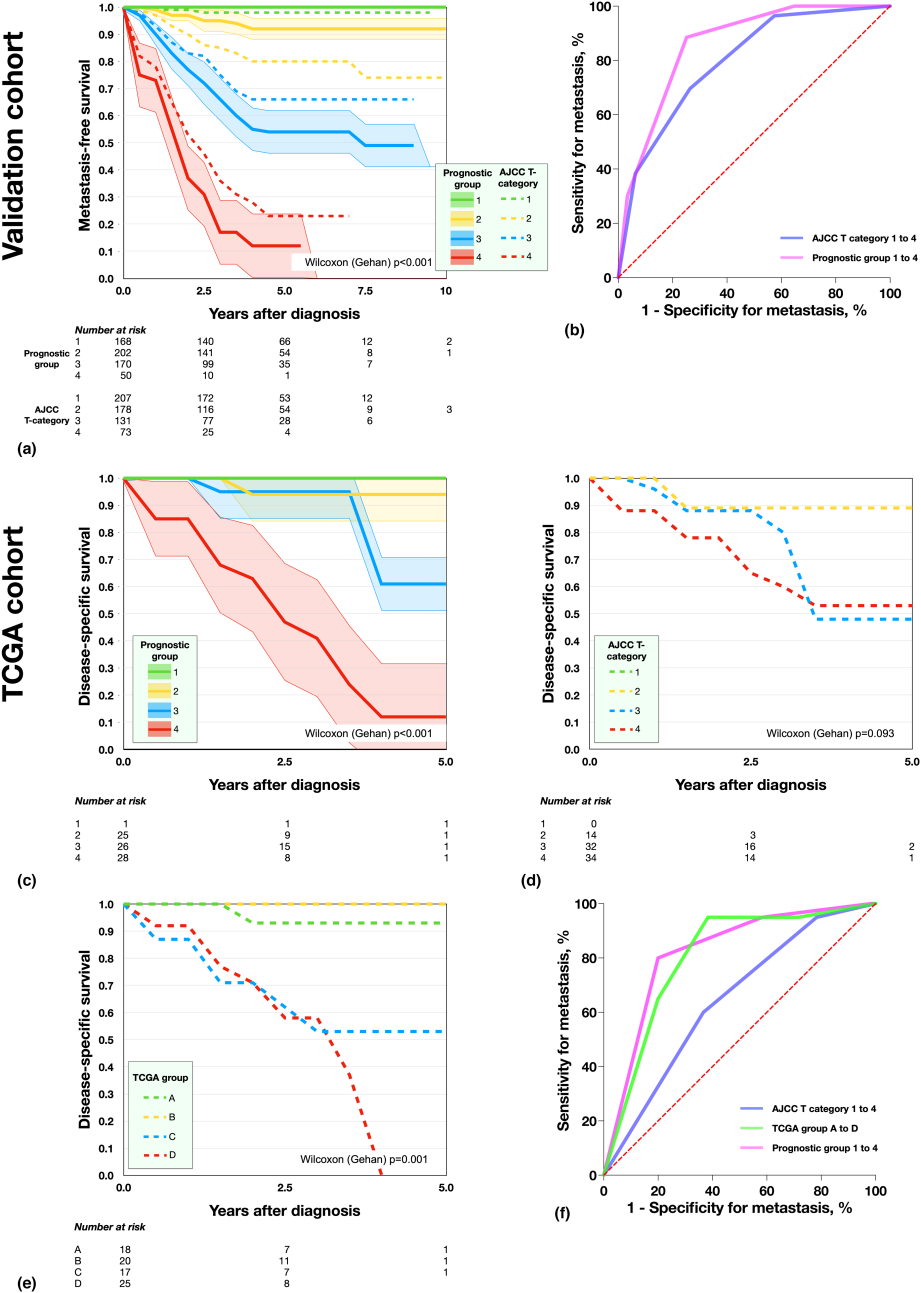
Prognostic group classification versus American Joint Committee on Cancer (AJCC) T category and the Cancer Genome Atlas (TCGA) groups A to D. (a) Survival according to prognostic group 1 (green), 2 (yellow), 3 (blue) and 4 (red) in a validation cohort consisting of a random sample from cohort 2. AJCC T categories 1–4 are included for comparison (dashed lines). (b) Receiver operating characteristics (ROC) curve of sensitivity and specificity for metastasis with the prognostic group classification and AJCC T categories 1–4. The prognostic groups achieved an AUC of 0.86 (95% CI 0.83–0.89) versus 0.80 for AJCC 4 T‐categories (95% CI 0.75–0.84). (c) Disease‐specific survival according to prognostic groups in the TCGA cohort. (d) Disease‐specific survival according to AJCC T‐category in the TCGA cohort. (e) Disease‐specific survival according to TCGA groups A to D in the TCGA cohort. (f) Prognostic groups achieved an AUC of 0.82 (95% CI 0.72 to 0.93) versus 0.80 for TCGA groups A to D (95% CI 0.70–0.91) and 0.64 for AJCC 4 T‐categories (95% CI 0.51–0.78). Ciliary body involvement. M3, Monosomy 3. AJCC, American Joint Committee on Cancer.

We then proceeded to test the prognostic point system in the validation cohort. Patients had gradually shorter metastasis‐free survival for each increased prognostic group (Wilcoxon (Gehan) *p* < 0.001, Figure 4a). Prognostic group 1 had no metastatic events at all, whereas the 5‐year metastasis‐free survival in group 4 was only 5%. In both univariate (Wald 161 vs. 118) and multivariate Cox regression analysis (Wald 54 vs. <1), the prognostic groups demonstrated greater acumen for the prediction of metastases than AJCC 4 T‐categories (Table 7). Further, in ROC, the prognostic groups achieved an AUC of 0.86 (95% CI 0.83–0.89) versus 0.80 for AJCC 4 T‐categories (95% CI 0.75–0.84, Figure 4b).

**TABLE 7:** Cox regression, hazard for metastasis in the validation cohort

	B	S.E.	Wald	P	Exp(B)	95% CI lower	95% CI upper
Validation cohort							
Univariate							
AJCC T‐category^a^	1.11	0.10	118.01	<0.001	3.03	2.48	3.70
Prognostic groups	1.68	0.13	161.03	<0.001	5.35	4.13	6.93
Multivariate							
AJCC T‐category^a^	0.08	0.17	0.23	0.63	1.08	0.78	1.51
Prognostic groups	1.59	0.22	54.29	<0.001	4.92	3.22	7.52
TCGA cohort							
Univariate							
AJCC T‐category^a^	0.72	0.38	3.56	0.059	2.05	0.97	4.34
TCGA groups A to D	1.08	0.29	14.15	<0.001	2.95	1.68	5.17
Prognostic groups	1.84	0.46	15.95	<0.001	6.28	2.55	15.48
Multivariate							
AJCC T‐category^a^	0.23	0.48	0.23	0.63	1.26	0.49	3.24
TCGA groups A to D	0.52	0.40	1.64	0.20	1.68	0.76	3.69
Prognostic groups	1.31	0.62	4.47	0.034	3.70	1.1	12.46

^a^ Size categories 1 through 4 without subclassification of CBI or extrascleral extension.

We also applied the prognostic point concept to the TCGA cohort and evaluated disease‐specific survival (proportion of patients not dead from metastatic UM). Again, patients had gradually shorter disease‐specific survival for each increased prognostic group (Wilcoxon (Gehan) *p* < 0.001, Figure 4c). No patient had AJCC T category 1 in this cohort and survival curves between T categories 2–4 did not differ significantly (*p* = 0.093, Figure 4d). Excellent separation between TCGA groups A to D was obtained (*p* < 0.001, Figure 4e). In univariate (Wald 16 vs. 14 vs. 4) and multivariate Cox regression analysis (Wald 4 vs. 2 vs. <1), prognostic groups demonstrated greater acumen for prediction of UM‐related mortality than TCGA groups A to D and AJCC 4 T‐categories, respectively (Table 7). Further, in ROC, the prognostic groups achieved an AUC of 0.82 (95% CI 0.72–0.93) versus 0.80 for TCGA groups A to D (95% CI 0.70–0.91) and 0.64 for AJCC 4 T‐categories (95% CI 0.51–0.78, Figure 4f).

## 4 DISCUSSION

In this study, we show that UMs with CBI are more common in women, have a greater apical thickness, greater basal tumour diameter, greater rates of vasculogenic mimicry, greater rates of M3 and poorer 5‐ and 10‐year globe retention rates. Tumours with CBI had significantly lower rates of retinal detachment and were more often asymptomatic at diagnosis. With increasing tumour diameter, thickness, or volume, a gradually larger proportion of tumours had M3, and a larger proportion of tumours with CBI had M3 at small diameters. Further, we confirm previous observations that CBI is an independent prognostic predictor in UM and that tumours with CBI are associated with M3, independent of patient age at diagnosis, tumour diameter and tumour thickness. Lastly, we show that an improved prognostic classification system can be proposed, based on patient age, sex, AJCC T‐categories and TCGA groups A to D. This classification system was shown to outperform the previous systems' ability to predict metastases. Other advantages include zero metastases in prognostic group 1, constituting 28% of the patient sample, which may be likened to zero false negatives for patients that are told that they are at low risk for metastasis. A very high metastatic rate in prognostic group 4 may be used when including patients in randomized trials with adjuvant treatment. Further, in analogy with TCGA groups A to D, the classification does not require any particularly complicated or expensive laboratory methods beyond the determination of copy numbers of chromosomes 3 and 8. The system is similar to the PRiMeUM and LUMPO III models for the prediction of metastatic risk, with the difference that our system assigns patients into one of four groups rather than provides individual risk estimations (Eleuteri et al., 2018; Vaquero‐Garcia et al., 2017). In contrast to PRiMeUM, our system includes extraocular extension and AJCC T category, but not chromosome 1p, 6p or 8p (Vaquero‐Garcia et al., 2017). LUMPO III also includes histological predictors, but not AJCC stage, T category or a stratification of 8q gains into 2, 3 or >3 copies (Eleuteri et al., 2018). It should be stressed that our comparison ignored the classification of AJCC a, b, c and d subcategories based on CBI and extraocular extension. As such, T categories could be described as a 17‐tiered system (T1 to T4 with subcategories a to d, and T4e) rather than a four‐tiered system. However, these 17 tiers should not be regarded as a continuum (e.g., T3a does not entail a worse prognosis than T2d), preventing their inclusion in regression analyses. Even if they were to be re‐coded into continuous variables, each increased step would likely entail a small increase in metastatic risk in relation to the four‐tiered prognostic groups. In this regard, AJCC stages I to IV might have been a more appropriate comparison, but it entails seven tiers (I, IIA, IIB, etc.) and takes systemic metastases into account.

Larger tumours are more likely to harbour many of the risk factors associated with worse outcomes including *BAP1* mutations, vasculogenic mimicry and M3 (Kim et al., 2019; Marathe et al., 2011; Szalai et al., 2018). Nevertheless, the prognostic importance of CBI bears witness to the appropriateness of adding CBI as a special characteristic when staging UM. This also indicates that there may be other factors than patient sex, age, tumour size and chromosome 3 status that explains the prognostic correlation. Other authors have suggested that the anatomical location or continuous contractions of the ciliary muscle or its rich vascularization may be causal factors to tumour spread (Costache et al., 2013; Kaliki & Shields, 2017). However, we are not aware of any observations that the vascularization of the ciliary body is richer than that of the choroid, or that causality between muscular contractions and metastatic seeding has been established. In 1996, Grossniklaus et al. (1996) observed that the metastatic rate was significantly lower in mice that had been inoculated with melanoma cells in the anterior chamber than in the posterior compartment. Rather, tumour infiltration of an adjacent tissue like the muscular bundles of the ciliary body may be regarded as a marker for invasive behaviour or ability, and CBI thus selects for a more aggressive group of tumours. Presumably, CBI may also be related to genetic aberrations that are not fully correlated to chromosome 3 status, such as DNA methylation events. Why such aggressive features would be more common when UMs with M3 involve the ciliary body remains unknown.

Further, the fact that CBI was more common in women while the risk for metastasis was higher in men is noteworthy. Previous studies have found similar results of higher rates of metastases in men (Rietschel et al., 2005; Zloto et al., 2013) whereas others have not (Shields et al., 2012; Stalhammar et al., 2019a, 2019b). Considering the strong prognostic value of CBI in this rather large cohort there may indeed be a protective factor associated with the female sex, or vice versa: A reason that more metastases develop in men regardless of patient age, tumour size, CBI and chromosome 3 status. Other authors have suggested that this may be related to differences in blood oestrogen levels or other hormonal factors (Holly et al., 1991; Zloto et al., 2013). The reader should be cautioned that competing risks were not accounted for.

Presenting symptoms differed significantly between the ciliary body and choroidal melanoma. One reason for the larger proportion of asymptomatic tumours with CBI is likely that more peripherally located tumours have to grow larger before they catch the attention of the patient. In turn, this might be a reason that tumours with CBI are diagnosed at a later stage when the tumour has reached larger dimensions. We have previously shown that patients presenting with a shadow in the visual field have significantly shorter disease‐specific survival, regardless of other symptoms, tumour size, location, local extent and stage (Fili et al., 2020). In turn, tumours from UM patients that report a visual field shadow are more likely to display vasculogenic mimicry and greater density of PAS positive patterns, which may underpin the association between this symptom and poor prognosis (Sabazade et al., 2021a, 2021b).

There are several limitations to this study. First and foremost, the data was retrospective and non‐randomized, which limits our control over confounding factors. Secondly, the number of patients with CBI was considerably smaller than the number of patients with choroidal melanoma. Although the proportional hazard assumption was met, we cannot exclude that this disproportionality skewed some of our statistical tests slightly. Thirdly, we had no data on death from other causes which would have allowed us to perform competing risk survival analyses instead of metastasis‐free and disease‐specific survival by an actuarial method and Cox regression. It is possible that our survival analyses were affected by competing risks, for example, that the survival differences related to patient sex were in fact biased by men dying at a greater rate from other diseases. Fourthly, data on tumour cell types, vasculogenic mimicry and retinal detachments were only available for a smaller proportion of patients. The results of comparisons of these factors may have been different if they were available for the full cohort. Fifthly, symptoms are subjective experiences. Some patients that reported a shadow may have experienced what others would describe as a floater, and the threshold for reporting a symptom likely differs significantly between individuals. And lastly, we neither had access to data on the *extent* of the ciliary body nor choroidal involvement. We only knew if the ciliary body was involved or not. It would have been highly interesting to examine if this extent has any prognostic implications.

In conclusion, primary UMs with CBI are associated with many poor prognostic factors. Along with CBI, male sex, patient age at diagnosis, tumour diameter and M3 are independent prognostic predictors for metastasis. A prognostic classification system that combines patient sex, age, AJCC 4 T categories and TCGA groups A to D is advantageous.

## Funding information

Support was provided to Dr. Stålhammar from:

- The Royal Swedish Academy of Sciences (reference ME2019‐0036)
- The Swedish Cancer Society (20 0798 Fk)
- The Swedish Society of Medicine (Cronqvists stiftelse, reference SLS 934014)
- The Swedish Eye Foundation (reference 2020‐04‐27)
- Karolinska Institutet (reference 2020‐013333 and 2020‐02517)
- Region Stockholm (reference 20200356).
- The Crown Princess Margareta Foundation for the Visually Impaired (reference 157)
- Carmen and Bertil Regnér Foundation (reference 2020‐00062)

## Supporting information


Figure S1

Figure S2

Table S1

Table S2

## Data Availability

The data that support the findings of this study are available from the corresponding author upon reasonable request. The results published here are in part based upon data generated by the TCGA Research Network: https://www. cancer.gov/tcga.

## References

[aos15210-bib-0001] Augsburger, J.J. & Gamel, J.W. (1990) Clinical prognostic factors in patients with posterior uveal malignant melanoma. Cancer, 66, 1596–1600. 2208011 10.1002/1097-0142(19901001)66:7<1596::aid-cncr2820660726>3.0.co;2-6

[aos15210-bib-0002] Berus, T. , Halon, A. , Markiewicz, A. , Orlowska‐Heitzman, J. , Romanowska‐Dixon, B. & Donizy, P. (2017) Clinical, histopathological and cytogenetic prognosticators in uveal melanoma – a comprehensive review. Anticancer Research, 37, 6541–6549. 29187428 10.21873/anticanres.12110

[aos15210-bib-0003] Costache, M. , Patrascu, O.M. , Adrian, D. , Costache, D. , Sajin, M. , Ungureanu, E. et al. (2013) Ciliary body melanoma – a particularly rare type of ocular tumor. Case report and general considerations. Maedica (Bucur), 8, 360–364. 24790669 PMC3968473

[aos15210-bib-0004] Damato, B. & Coupland, S.E. (2009) A reappraisal of the significance of largest basal diameter of posterior uveal melanoma. Eye (London, England), 23, 2152–2160 quiz 2161‐2152. 19876071 10.1038/eye.2009.235

[aos15210-bib-0005] Eleuteri, A. , Taktak, A.F.G. , Coupland, S.E. , Heimann, H. , Kalirai, H. & Damato, B. (2018) Prognostication of metastatic death in uveal melanoma patients: A Markov multi‐state model. Computers in Biology and Medicine, 102, 151–156. 30278339 10.1016/j.compbiomed.2018.09.024

[aos15210-bib-0006] Ewens, K.G. , Kanetsky, P.A. , Richards‐Yutz, J. , Al‐Dahmash, S. , De Luca, M.C. , Bianciotto, C.G. et al. (2013) Genomic profile of 320 uveal melanoma cases: chromosome 8p‐loss and metastatic outcome. Investigative Ophthalmology & Visual Science, 54, 5721–5729. 23821189 10.1167/iovs.13-12195

[aos15210-bib-0007] Field, M.G. , Durante, M.A. , Anbunathan, H. , Cai, L.Z. , Decatur, C.L. , Bowcock, A.M. et al. (2018) Punctuated evolution of canonical genomic aberrations in uveal melanoma. Nature Communications, 9, 116. 10.1038/s41467-017-02428-w PMC5760704 29317634

[aos15210-bib-0008] Fili, M. , Seregard, S. & Stalhammar, G. (2020) Presenting symptoms are associated with uveal melanoma‐related death. Ophthalmology, 128, 1107–1109. 33253758 10.1016/j.ophtha.2020.11.023

[aos15210-bib-0009] Folberg, R. , Pe'Er, J. , Gruman, L.M. , Woolson, R.F. , Jeng, G. , Montague, P.R. et al. (1992) The morphologic characteristics of tumor blood vessels as a marker of tumor progression in primary human uveal melanoma: a matched case‐control study. Human Pathology, 23, 1298–1305. 1427757 10.1016/0046-8177(92)90299-i

[aos15210-bib-0010] Gelmi, M.C. , Bas, Z. , Malkani, K. , Ganguly, A. , Shields, C.L. & Jager, M.J. (2022) Adding the cancer genome atlas chromosome classes to American joint committee on cancer system offers more precise prognostication in uveal melanoma. Ophthalmology, 129, 431–437. 34793831 10.1016/j.ophtha.2021.11.018

[aos15210-bib-0011] Grossniklaus, H.E. , Wilson, M.W. , Barron, B.C. & Lynn, M.J. (1996) Anterior vs posterior intraocular melanoma. Metastatic differences in a murine model. Archives of Ophthalmology, 114, 1116–1120. 8790099 10.1001/archopht.1996.01100140318011

[aos15210-bib-0012] Harbour, J.W. , Onken, M.D. , Roberson, E.D. , Duan, S. , Cao, L. , Worley, L.A. et al. (2010) Frequent mutation of BAP1 in metastasizing uveal melanomas. Science, 330, 1410–1413. 21051595 10.1126/science.1194472 PMC3087380

[aos15210-bib-0013] Harbour, J.W. , Roberson, E.D. , Anbunathan, H. , Onken, M.D. , Worley, L.A. & Bowcock, A.M. (2013) Recurrent mutations at codon 625 of the splicing factor SF3B1 in uveal melanoma. Nature Genetics, 45, 133–135. 23313955 10.1038/ng.2523 PMC3789378

[aos15210-bib-0014] Holly, E.A. , Aston, D.A. , Ahn, D.K. , Kristiansen, J.J. & Char, D.H. (1991) Uveal melanoma, hormonal and reproductive factors in women. Cancer Research, 51, 1370–1372. 1997174

[aos15210-bib-0015] Horsman, D.E. & White, V.A. (1993) Cytogenetic analysis of uveal melanoma. Consistent occurrence of monosomy 3 and trisomy 8q. Cancer, 71, 811–819. 8431862 10.1002/1097-0142(19930201)71:3<811::aid-cncr2820710325>3.0.co;2-f

[aos15210-bib-0016] Jager, M.J. , Brouwer, N.J. & Esmaeli, B. (2018) The cancer genome atlas project: an integrated molecular view of uveal melanoma. Ophthalmology, 125, 1139–1142. 30032793 10.1016/j.ophtha.2018.03.011

[aos15210-bib-0017] Jager, M.J. , Shields, C.L. , Cebulla, C.M. , Abdel‐Rahman, M.H. , Grossniklaus, H.E. , Stern, M.H. et al. (2020) Uveal melanoma. Nature Reviews. Disease Primers, 6, 24. 10.1038/s41572-020-0158-0 32273508

[aos15210-bib-0018] Johansson, P.A. , Brooks, K. , Newell, F. , Palmer, J.M. , Wilmott, J.S. , Pritchard, A.L. et al. (2020) Whole genome landscapes of uveal melanoma show an ultraviolet radiation signature in iris tumours. Nature Communications, 11, 2408. 10.1038/s41467-020-16276-8 PMC7229209 32415113

[aos15210-bib-0019] Kaliki, S. & Shields, C.L. (2017) Uveal melanoma: relatively rare but deadly cancer. Eye (London, England), 31, 241–257. 27911450 10.1038/eye.2016.275 PMC5306463

[aos15210-bib-0020] Kim, H.S. , Won, Y.J. , Shim, J.H. , Kim, H.J. , Kim, J. , Hong, H.N. et al. (2019) Morphological characteristics of vasculogenic mimicry and its correlation with EphA2 expression in gastric adenocarcinoma. Scientific Reports, 9, 3414. 30833656 10.1038/s41598-019-40265-7 PMC6399224

[aos15210-bib-0021] Kujala, E. , Damato, B. , Coupland, S.E. , Desjardins, L. , Bechrakis, N.E. , Grange, J.D. et al. (2013) Staging of ciliary body and choroidal melanomas based on anatomic extent. Journal of Clinical Oncology, 31, 2825–2831. 23816968 10.1200/JCO.2012.45.2771

[aos15210-bib-0022] Kujala, E. & Kivela, T. (2005) Tumor, node, metastasis classification of malignant ciliary body and choroidal melanoma evaluation of the 6th edition and future directions. Ophthalmology, 112, 1135–1144. 15885792 10.1016/j.ophtha.2004.11.063

[aos15210-bib-0023] Li, W. , Gragoudas, E.S. & Egan, K.M. (2000) Metastatic melanoma death rates by anatomic site after proton beam irradiation for uveal melanoma. Archives of Ophthalmology, 118, 1066–1070. 10922199 10.1001/archopht.118.8.1066

[aos15210-bib-0024] Makitie, T. , Summanen, P. , Tarkkanen, A. & Kivela, T. (1999) Microvascular loops and networks as prognostic indicators in choroidal and ciliary body melanomas. Journal of the National Cancer Institute, 91, 359–367. 10050870 10.1093/jnci/91.4.359

[aos15210-bib-0025] Marathe, O.S. , Wu, J. , Lee, S.P. , Yu, F. , Burgess, B.L. , Leu, M. et al. (2011) Ocular response of choroidal melanoma with monosomy 3 versus disomy 3 after iodine‐125 brachytherapy. International Journal of Radiation Oncology, Biology, Physics, 81, 1046–1048. 20932685 10.1016/j.ijrobp.2010.07.016

[aos15210-bib-0026] Martin, M. , Masshofer, L. , Temming, P. , Rahmann, S. , Metz, C. , Bornfeld, N. et al. (2013) Exome sequencing identifies recurrent somatic mutations in EIF1AX and SF3B1 in uveal melanoma with disomy 3. Nature Genetics, 45, 933–936. 23793026 10.1038/ng.2674 PMC4307600

[aos15210-bib-0027] Mazloumi, M. , Vichitvejpaisal, P. , Dalvin, L.A. , Yaghy, A. , Ewens, K.G. , Ganguly, A. et al. (2020) Accuracy of the cancer genome atlas classification vs American joint committee on cancer classification for prediction of metastasis in patients with uveal melanoma. JAMA Ophthalmology, 138, 260–267. 31944225 10.1001/jamaophthalmol.2019.5710 PMC6990957

[aos15210-bib-0028] McLean, I.W. , Foster, W.D. & Zimmerman, L.E. (1982) Uveal melanoma: location, size, cell type, and enucleation as risk factors in metastasis. Human Pathology, 13, 123–132. 7076200 10.1016/s0046-8177(82)80116-0

[aos15210-bib-0029] McLean, I.W. , Keefe, K.S. & Burnier, M.N. (1997) Uveal melanoma. Comparison of the prognostic value of fibrovascular loops, mean of the ten largest nucleoli, cell type, and tumor size. Ophthalmology, 104, 777–780. 9160022 10.1016/s0161-6420(97)30234-6

[aos15210-bib-0030] Meir, T. , Zeschnigk, M. , Masshofer, L. , Pe'er, J. & Chowers, I. (2007) The spatial distribution of monosomy 3 and network vasculogenic mimicry patterns in uveal melanoma. Investigative Ophthalmology & Visual Science, 48, 1918–1922. 17460242 10.1167/iovs.06-1308

[aos15210-bib-0031] Onken, M.D. , Worley, L.A. , Ehlers, J.P. & Harbour, J.W. (2004) Gene expression profiling in uveal melanoma reveals two molecular classes and predicts metastatic death. Cancer Research, 64, 7205–7209. 15492234 10.1158/0008-5472.CAN-04-1750 PMC5407684

[aos15210-bib-0032] Onken, M.D. , Worley, L.A. , Long, M.D. , Duan, S. , Council, M.L. , Bowcock, A.M. et al. (2008) Oncogenic mutations in GNAQ occur early in uveal melanoma. Investigative Ophthalmology & Visual Science, 49, 5230–5234. 18719078 10.1167/iovs.08-2145 PMC2634606

[aos15210-bib-0033] Park, S.J. , Oh, C.M. , Yeon, B. , Cho, H. & Park, K.H. (2017) Sex disparity in survival of patients with uveal melanoma: better survival rates in women than in men in South Korea. Investigative Ophthalmology & Visual Science, 58, 1909–1915. 28362882 10.1167/iovs.16-20077

[aos15210-bib-0034] Prescher, G. , Bornfeld, N. & Becher, R. (1990) Nonrandom chromosomal abnormalities in primary uveal melanoma. Journal of the National Cancer Institute, 82, 1765–1769. 2231772 10.1093/jnci/82.22.1765

[aos15210-bib-0035] Prescher, G. , Bornfeld, N. , Hirche, H. , Horsthemke, B. , Jockel, K.H. & Becher, R. (1996) Prognostic implications of monosomy 3 in uveal melanoma. Lancet, 347, 1222–1225. 8622452 10.1016/s0140-6736(96)90736-9

[aos15210-bib-0036] Rietschel, P. , Panageas, K.S. , Hanlon, C. , Patel, A. , Abramson, D.H. & Chapman, P.B. (2005) Variates of survival in metastatic uveal melanoma. Journal of Clinical Oncology, 23, 8076–8080. 16258106 10.1200/JCO.2005.02.6534

[aos15210-bib-0037] Robertson, A.G. , Shih, J. , Yau, C. , Gibb, E.A. , Oba, J. , Mungall, K.L. et al. (2017) Integrative analysis identifies four molecular and clinical subsets in uveal melanoma. Cancer Cell, 32, 204–220.e215. 28810145 10.1016/j.ccell.2017.07.003 PMC5619925

[aos15210-bib-0038] Rodrigues, M. , Ait Rais, K. , Salviat, F. , Algret, N. , Simaga, F. , Barnhill, R. et al. (2020) Association of Partial Chromosome 3 deletion in uveal melanomas with metastasis‐free survival. JAMA Ophthalmology, 138, 182–188. 31895446 10.1001/jamaophthalmol.2019.5403 PMC6990836

[aos15210-bib-0039] Rummelt, V. , Folberg, R. , Woolson, R.F. , Hwang, T. & Pe'er, J. (1995) Relation between the microcirculation architecture and the aggressive behavior of ciliary body melanomas. Ophthalmology, 102, 844–851. 7777286 10.1016/s0161-6420(95)30947-5

[aos15210-bib-0040] Sabazade, S. , Gill, V. , Herrspiegel, C. & Stalhammar, G. (2021a) Vasculogenic mimicry correlates to presenting symptoms and mortality in uveal melanoma. Journal of Cancer Research and Clinical Oncology, 148, 587–597. 34775516 10.1007/s00432-021-03851-9 PMC8881423

[aos15210-bib-0041] Sabazade, S. , Herrspiegel, C. , Gill, V. & Stålhammar, G. (2021b) No differences in the long‐term prognosis of iris and choroidal melanomas when adjusting for tumor thickness and diameter. BMC Cancer, 21, 1–8. 34819035 10.1186/s12885-021-09002-0 PMC8614046

[aos15210-bib-0042] Schmitzer, S. , Butea‐Simionescu, C. , Gheorghe, A. , Zemba, M. & Cioboata, M. (2016) Iridociliary melanoma ‐ Clinical case. Journal of Medicine and Life, 9, 88–91. 27713771 PMC5052490

[aos15210-bib-0043] Scholes, A.G. , Damato, B.E. , Nunn, J. , Hiscott, P. , Grierson, I. & Field, J.K. (2003) Monosomy 3 in uveal melanoma: correlation with clinical and histologic predictors of survival. Investigative Ophthalmology & Visual Science, 44, 1008–1011. 12601021 10.1167/iovs.02-0159

[aos15210-bib-0044] Seddon, J.M. , Albert, D.M. , Lavin, P.T. & Robinson, N. (1983) A prognostic factor study of disease‐free interval and survival following enucleation for uveal melanoma. Archives of Ophthalmology, 101, 1894–1899. 6651594 10.1001/archopht.1983.01040020896012

[aos15210-bib-0045] Seregard, S. , Spångberg, B. , Juul, C. & Oskarsson, M. (1998) Prognostic accuracy of the mean of the largest nucleoli, vascular patterns, and PC‐10 in posterior uveal melanoma. Ophthalmology, 105, 485–491. 9499780 10.1016/S0161-6420(98)93032-9

[aos15210-bib-0046] Shields, C.L. , Furuta, M. , Thangappan, A. , Nagori, S. , Mashayekhi, A. , Lally, D.R. et al. (2009) Metastasis of uveal melanoma millimeter‐by‐millimeter in 8033 consecutive eyes. Archives of Ophthalmology, 127, 989–998. 19667335 10.1001/archophthalmol.2009.208

[aos15210-bib-0047] Shields, C.L. , Ganguly, A. , Materin, M.A. , Teixeira, L. , Mashayekhi, A. , Swanson, L.A. et al. (2007) Chromosome 3 analysis of uveal melanoma using fine‐needle aspiration biopsy at the time of plaque radiotherapy in 140 consecutive cases: the Deborah Iverson, MD, lectureship. Archives of Ophthalmology, 125, 1017–1024. 17698747 10.1001/archopht.125.8.1017

[aos15210-bib-0048] Shields, C.L. , Kaliki, S. , Furuta, M. , Mashayekhi, A. & Shields, J.A. (2012) Clinical spectrum and prognosis of uveal melanoma based on age at presentation in 8,033 cases. Retina, 32, 1363–1372. 22466491 10.1097/IAE.0b013e31824d09a8

[aos15210-bib-0049] Shields, C.L. , Say, E.A.T. , Hasanreisoglu, M. , Saktanasate, J. , Lawson, B.M. , Landy, J.E. et al. (2017a) Personalized prognosis of uveal melanoma based on cytogenetic profile in 1059 patients over an 8‐year period: the 2017 Harry S. Gradle Lecture. Ophthalmology, 124, 1523–1531. 28495150 10.1016/j.ophtha.2017.04.003

[aos15210-bib-0050] Shields, C.L. , Say, E.A.T. , Hasanreisoglu, M. , Saktanasate, J. , Lawson, B.M. , Landy, J.E. et al. (2017b) Cytogenetic abnormalities in uveal melanoma based on tumor features and size in 1059 patients: the 2016 W. Richard Green Lecture. Ophthalmology, 124, 609–618. 28159380 10.1016/j.ophtha.2016.12.026

[aos15210-bib-0051] Simpson, E.R. , Gallie, B. , Saakyan, S. , Amiryan, A. , Finger, P.T. , Chin, K.J. et al. (2015) International validation of the American joint committee on Cancer's 7th edition classification of uveal melanoma. JAMA Ophthalmology, 133, 376–383. 25555246 10.1001/jamaophthalmol.2014.5395

[aos15210-bib-0052] Sisley, K. , Rennie, I.G. , Cottam, D.W. , Potter, A.M. , Potter, C.W. & Rees, R.C. (1990) Cytogenetic findings in six posterior uveal melanomas: involvement of chromosomes 3, 6, and 8. Genes, Chromosomes & Cancer, 2, 205–209. 2078511 10.1002/gcc.2870020307

[aos15210-bib-0053] Sisley, K. , Rennie, I.G. , Parsons, M.A. , Jacques, R. , Hammond, D.W. , Bell, S.M. et al. (1997) Abnormalities of chromosomes 3 and 8 in posterior uveal melanoma correlate with prognosis. Genes, Chromosomes & Cancer, 19, 22–28. 9135991 10.1002/(sici)1098-2264(199705)19:1<22::aid-gcc4>3.0.co;2-2

[aos15210-bib-0054] Stalhammar, G. , See, T.R. , Fili, M. & Seregard, S. (2019a) No gender differences in Long‐term survival after brachytherapy of 1,541 patients with uveal melanoma. Ocular Oncology and Pathology, 5, 432–439. 31768367 10.1159/000497186 PMC6873032

[aos15210-bib-0055] Stalhammar, G. , See, T.R.O. , Phillips, S.S. & Grossniklaus, H.E. (2019b) Density of PAS positive patterns in uveal melanoma: correlation with vasculogenic mimicry, gene expression class, BAP‐1 expression, macrophage infiltration, and risk for metastasis. Molecular Vision, 25, 502–516. 31588174 PMC6776441

[aos15210-bib-0056] Szalai, E. , Wells, J.R. , Ward, L. & Grossniklaus, H.E. (2018) Uveal melanoma nuclear BRCA1‐associated Protein‐1 immunoreactivity is an indicator of metastasis. Ophthalmology, 125, 203–209. 28823399 10.1016/j.ophtha.2017.07.018 PMC6173805

[aos15210-bib-0057] Torossian, N.M. , Wallace, R.T. , Hwu, W.J. & Bedikian, A.Y. (2015) Metastasis of ciliary body melanoma to the contralateral eye: a case report and review of uveal melanoma literature. Case Reports in Oncological Medicine, 2015, 427163. 25874144 10.1155/2015/427163 PMC4385596

[aos15210-bib-0058] Uner, O.E. , See, T.R.O. , Szalai, E. , Grossniklaus, H.E. & Stalhammar, G. (2021) Estimation of the timing of BAP1 mutation in uveal melanoma progression. Scientific Reports, 11, 8923. 33903674 10.1038/s41598-021-88390-6 PMC8076235

[aos15210-bib-0059] van Beek, J.G. , Koopmans, A.E. , Vaarwater, J. , de Rooi, J.J. , Paridaens, D. , Naus, N.C. et al. (2014) The prognostic value of extraocular extension in relation to monosomy 3 and gain of chromosome 8q in uveal melanoma. Investigative Ophthalmology & Visual Science, 55, 1284–1291. 24508790 10.1167/iovs.13-13670

[aos15210-bib-0060] Van Raamsdonk, C.D. , Bezrookove, V. , Green, G. , Bauer, J. , Gaugler, L. , O'Brien, J.M. et al. (2009) Frequent somatic mutations of GNAQ in uveal melanoma and blue naevi. Nature, 457, 599–602. 19078957 10.1038/nature07586 PMC2696133

[aos15210-bib-0061] Van Raamsdonk, C.D. , Griewank, K.G. , Crosby, M.B. , Garrido, M.C. , Vemula, S. , Wiesner, T. et al. (2010) Mutations in GNA11 in uveal melanoma. The New England Journal of Medicine, 363, 2191–2199. 21083380 10.1056/NEJMoa1000584 PMC3107972

[aos15210-bib-0062] Vaquero‐Garcia, J. , Lalonde, E. , Ewens, K.G. , Ebrahimzadeh, J. , Richard‐Yutz, J. , Shields, C.L. et al. (2017) PRiMeUM: a model for predicting risk of metastasis in uveal melanoma. Investigative Ophthalmology & Visual Science, 58, 4096–4105. 28828481 10.1167/iovs.17-22255 PMC6108308

[aos15210-bib-0063] von Elm, E. , Altman, D.G. , Egger, M. , Pocock, S.J. , Gotzsche, P.C. , Vandenbroucke, J.P. et al. (2007) The strengthening the reporting of observational studies in epidemiology (STROBE) statement: guidelines for reporting observational studies. Lancet, 370, 1453–1457. 18064739 10.1016/S0140-6736(07)61602-X

[aos15210-bib-0064] Worley, L.A. , Onken, M.D. , Person, E. , Robirds, D. , Branson, J. , Char, D.H. et al. (2007) Transcriptomic versus chromosomal prognostic markers and clinical outcome in uveal melanoma. Clinical Cancer Research, 13, 1466–1471. 17332290 10.1158/1078-0432.CCR-06-2401

[aos15210-bib-0065] Zloto, O. , Pe'er, J. & Frenkel, S. (2013) Gender differences in clinical presentation and prognosis of uveal melanoma. Investigative Ophthalmology & Visual Science, 54, 652–656. 23197684 10.1167/iovs.12-10365

