# Supplementary figures and images for: A prognostic classification system for uveal melanoma based on a combination of patient age and sex, the American Joint Committee on Cancer and the Cancer Genome Atlas models
Source: Acta Ophthalmol. 2022 Jul 8;101(1):34–48. doi: 10.1111/aos.15210 (PMC10083913; doi:10.1111/aos.15210)

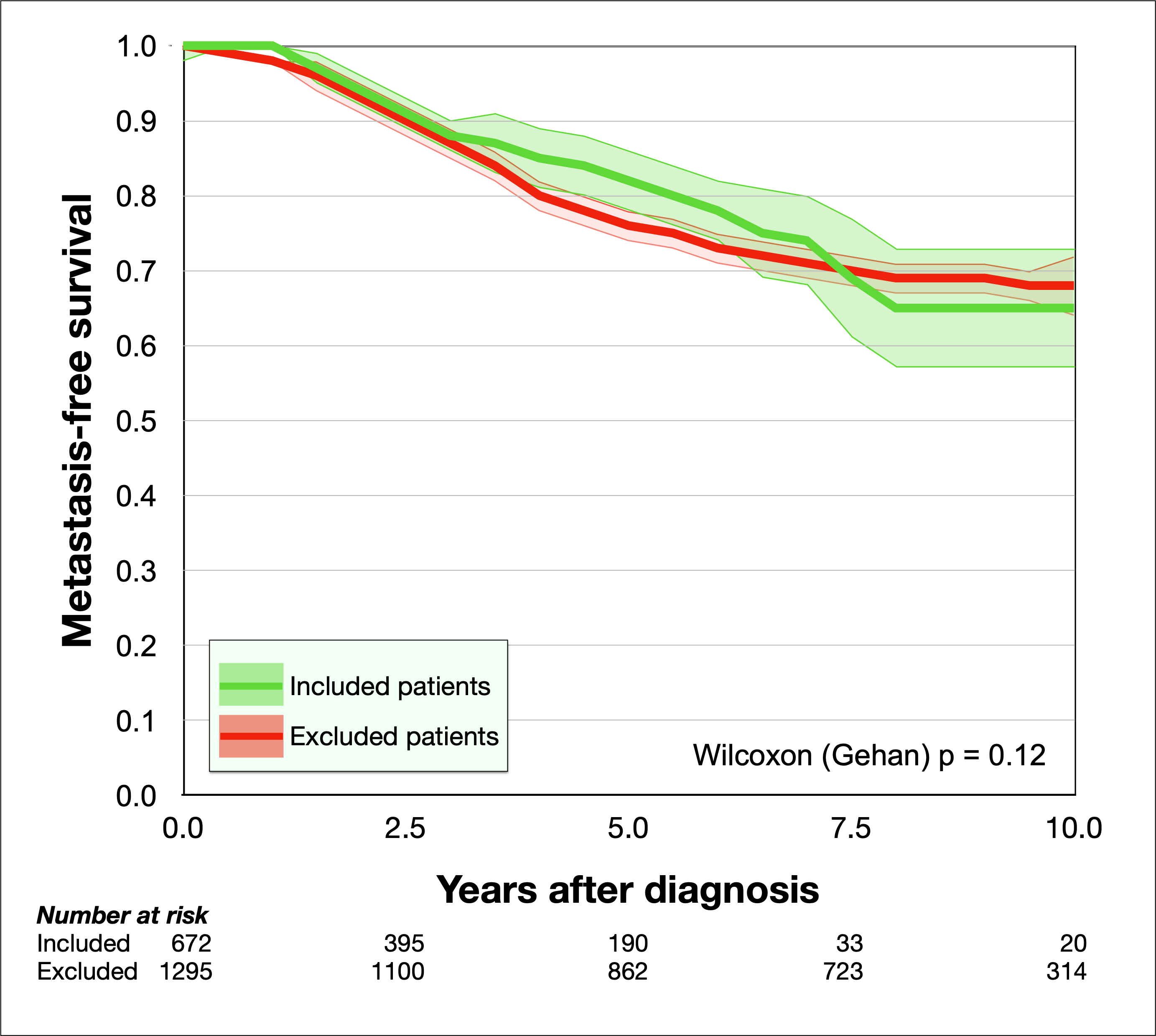

Supplement: Supplementary file 1 — Figure S1 [file AOS-101-34-s004.tiff]

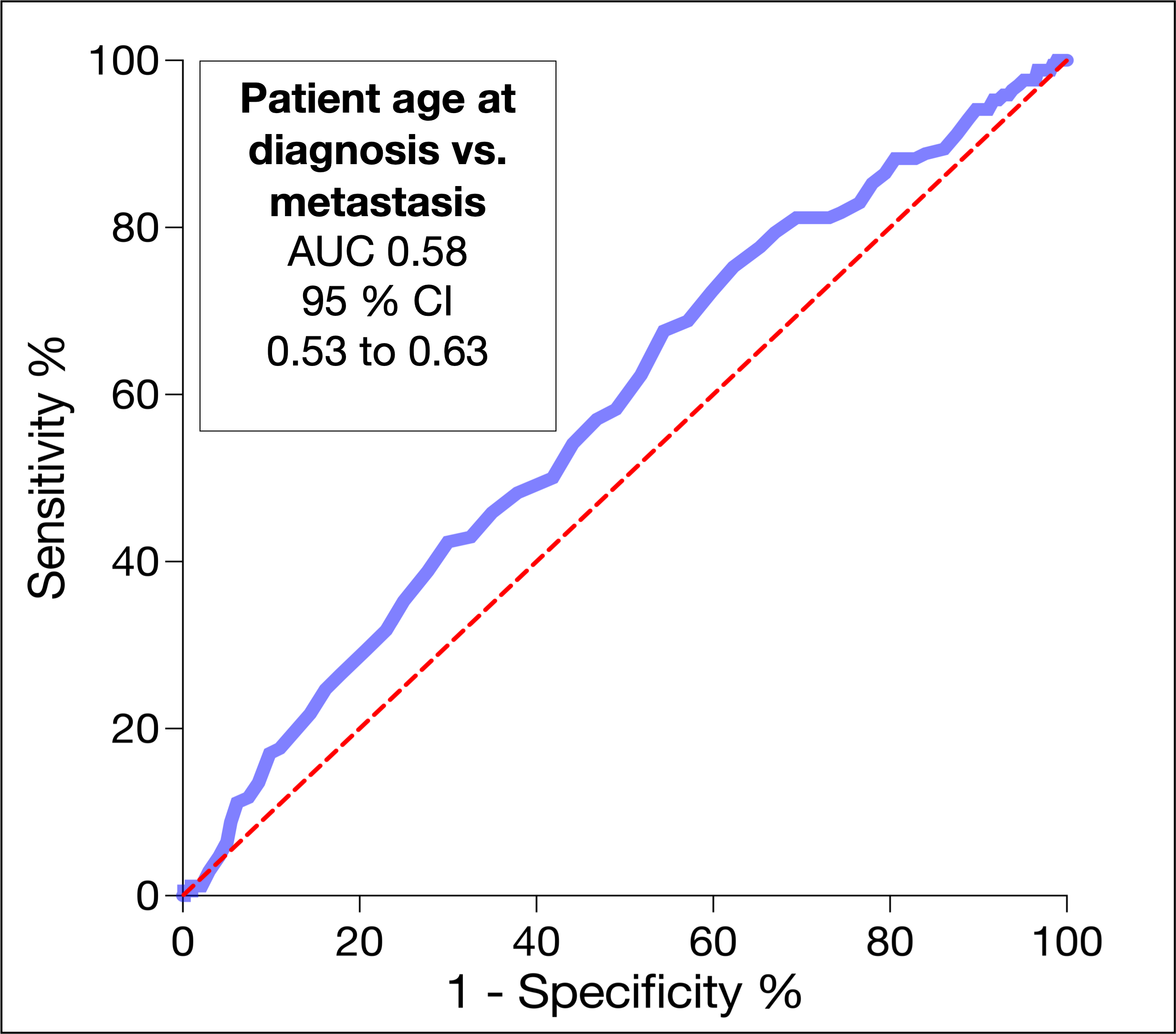

Supplement: Supplementary file 2 — Figure S2 [file AOS-101-34-s003.tiff]
