# Supplementary material for: A prognostic classification system for uveal melanoma based on a combination of patient age and sex, the American Joint Committee on Cancer and the Cancer Genome Atlas models
Source: Acta Ophthalmol. 2022 Jul 8;101(1):34–48. doi: 10.1111/aos.15210 (PMC10083913; doi:10.1111/aos.15210)
Supplement: Supplementary file 3 — Table S1 [file AOS-101-34-s002.pdf]

Supplementary table 1. Summary of the included cohorts.		
**Variable**	**Cohort 1**	**Cohort 2**
n	672	1124
Patient gender	✅	✅
Patient age at diagnosis	✅	✅
Tumor diameter	✅	✅
Tumor thickness	✅	✅
Ciliary body involvement yes/no	✅	✅
AJCC T-category	✅	✅
Follow-up incl. metastases	✅	✅
Chromosome 3 status	🚫	✅
Presenting symptoms	✅	🚫
Primary tumor treatment	✅	🚫
Treatment failure and secondary enucleation	✅	🚫
Vasculogenic mimicry (for 116 enucleated tumors)	✅	🚫

✅ Data available. 🚫 Data not available.
