# Supplementary material for: A prognostic classification system for uveal melanoma based on a combination of patient age and sex, the American Joint Committee on Cancer and the Cancer Genome Atlas models
Source: Acta Ophthalmol. 2022 Jul 8;101(1):34–48. doi: 10.1111/aos.15210 (PMC10083913; doi:10.1111/aos.15210)
Supplement: Supplementary file 4 — Table S2 [file AOS-101-34-s001.pdf]

Supplementary table 2. Cox regression, hazard for metastasis in training cohort							
	**B**	**S.E.**	**Wald**	**P**	**Exp(B)**	**95 % CI lower**	**95 % CI upper**
**Multivariate**							
Male sex	0.51	0.19	7.20	0.007	1.66	1.15	2.40
Age at diagnosis[a]	0.19	0.07	7.91	0.005	1.21	1.06	1.38
AJCC T-category[b]	0.98	0.11	82.14	<0.001	2.66	2.15	3.28
Monosomy 3	0.91	0.23	16.22	<0.001	2.48	1.59	3.86
CBI	0.48	0.20	5.81	0.016	1.62	1.09	2.40

[a]Per increased decade. [b]Size category 1 through 4 without subclassification of CBI or extraocular extension. CBI, Ciliary body involvement.
